# Diffusion Weighted MRI for Hepatic Fibrosis: Impact of b-Value

**DOI:** 10.5812/iranjradiol.3555

**Published:** 2014-01-30

**Authors:** Huseyin Ozkurt, Firat Keskiner, Ozan Karatag, Canan Alkim, Sukru Mehmet Erturk, Muzaffer Basak

**Affiliations:** 1 Department of Radiology, Sisli Etfal Education and Research Hospital, Istanbul, Turkey; 2 Department of Radiology, Canakkale Onsekiz Mart University, Faculty of Medicine, Canakkale, Turkey; 3 Department of Gastroenterology, Sisli Etfal Education and Research Hospital, Istanbul, Turkey

**Keywords:** Liver Cirrhosis, Magnetic Resonance Imaging, Diffusion Magnetic Resonance Imaging

## Abstract

**Background:**

Hepatic fibrosis is a typical complication of chronic liver diseases resulting in cirrhosis that remains a major public health problem worldwide. Liver biopsy is currently the gold standard for diagnosing and staging hepatic fibrosis. Percutaneous liver biopsy; however, is an invasive procedure with risks of complications. Therefore, there is need for alternative non-invasive techniques to assess liver fibrosis and chronic liver diseases. In recent years, MRI techniques, including diffusion weighted imaging (DWI), have been developed for in vivo quantification of liver fibrosis.

**Objectives:**

The purpose of this study is to evaluate the utility of diffusion weighted MRI in the diagnosis and quantification of the degree of hepatic fibrosis and to investigate the influence of *b*-value.

**Patients and Methods:**

Twenty-four patients (13 males, 11 females), with a mean age of 46 years (36-73 years) diagnosed as chronic hepatitis and histopathologically proven liver fibrosis and 22 other patients (8 males, 14 females) with no clinical or biochemical findings of liver disease, with a mean age of 51.2 years (32-75 years) were included in the study. All patients with chronic hepatitis underwent percutaneous liver biopsy by an experienced hepatologist without sonographic guidance. The Knodell histology activity index (HAI) for grading of necroinflammatory changes and Metavir scoring system for staging of the liver fibrosis were used to record the severity of the disease. All patients were examined with a 1.5 Tesla MRI system and the patients underwent diffusion weighted imaging (DWI) with a routine hepatic MRI protocol. Different *b*-values including 250, 500, 750, and 1000 sec/mm ^2^ were used to calculate apparent diffusion coefficients.

**Results:**

We detected decreased apparent diffusion coefficient values in patients with hepatic fibrosis compared to patients without chronic hepatitis and there was a trend toward decrease in hepatic apparent diffusion coefficient values with an increasing degree of fibrosis.

**Conclusions:**

Our findings suggest that hepatic apparent diffusion coefficient measurement with a *b*-value of 750 sec/mm ^2^ or greater is useful in accurate quantification of liver fibrosis and necroinflammation.

## 1. Background

Hepatic fibrosis is a typical complication of chronic liver diseases resulting in cirrhosis that remains a major public health problem worldwide (1, 2). Several factors have been described as being involved in the development of cirrhosis. These factors are mainly alcohol ingestion and hepatitis B and C infections that are stated as 80-90% of the cases in the literature. Other causes are hemochromatosis, primary biliary cirrhosis, primary sclerosing cholangitis, Wilson’s disease, autoimmune disease, Budd-Chiari syndrome and nonalcoholic steatohepatitis (3). Liver biopsy is currently the gold standard for diagnosing and staging hepatic fibrosis (1, 2, 4-6). Percutaneous liver biopsy; however, is an invasive procedure with risks of complications such as pain, hemorrhage, bile peritonitis, penetration to abdominal viscera, pneumothorax and death (6, 7). This procedure is also prone to interobserver variability and sampling error (1, 4, 8-11). In the literature, different morbidity and mortality rates have been mentioned for this invasive procedure. Tobkes et al. (7) stated that needle biopsy of the liver has a mortality rate between 0.009% and 0.12%; while in Wong et al.’s study (12), an 0.018% rate was declared. In addition, in a study conducted by Piccinino et al. (13), a morbidity of 3% and a mortality of 0.03% were pointed out for percutaneous liver biopsy (13). In another study, a 24% estimated false negative result was notified for percutaneous liver biopsy due to inter-observer variability and sampling errors (14). Therefore, alternative non-invasive techniques have been developed to assess liver fibrosis and chronic liver diseases. Unfortunately, these approaches including routine biochemical and hematological tests, serum markers of connective tissue, and scoring systems using a combination of clinical and/or laboratory tests (4, 15-20) are not sensitive and specific enough to quantify liver fibrosis (2). Recently, measurement of liver stiffness with ultrasound transient elastography has been validated to detect significant fibrosis in patients with chronic hepatitis C. However, this method cannot be applied to patients with ascites, narrow intercostal spaces and overweight status (4, 21).

In recent years, magnetic resonance imaging (MRI) techniques, including diffusion weighted imaging (DWI), have been developed for in vivo quantification of liver fibrosis (4, 22). This is an imaging method in which the severity of the disease is quantified by combined effects of capillary perfusion and diffusion using apparent diffusion coefficient (ADC) measurement (22-24). Several reports suggest that measures of DWI show lower ADC values in cirrhotic livers than in normal livers (2, 4, 22, 25, 26). This is due to accumulation of fibrosis leading to reduction in the amount of water proton diffusion in the affected liver tissue (1, 2, 27).

## 2. Objectives

The aim of our study is to evaluate the potential role of DWI in diagnosing the presence and quantifying the degree of hepatic fibrosis. 

## 3. Patients and Methods

### 3.1. Patient Selection

The protocol for this study was approved by our ethics committee. Twenty-four patients (13 males, 11 females), with a mean age of 46 years (range: 36-73 years) diagnosed with chronic hepatitis and histopathologically proven liver fibrosis and 22 other patients with no clinical or biochemical findings for liver disease (8 males, 14 females), with a mean age of 51.2 years (range: 32-75 years) were included in the study. Liver disease was diagnosed on the basis of clinical history, liver function test results, and percutaneous liver biopsy that was clinically indicated. The causes of liver disease were chronic hepatitis C virus infection (n=15), chronic hepatitis B virus infection (n=8), and chronic hepatitis B+D virus infection (n=1). None of the patients had a diagnosis of hepatocellular carcinoma. Patients with focal malignant lesions of the liver visible on imaging procedures including ultrasound, computed tomography (CT) or MRI were excluded from the study. The patients without liver disease had neither a previous history of liver disease nor alcohol abuse.

### 3.2. Histopathologic Assessment

All patients underwent percutaneous liver biopsy by an experienced hepatologist using a 20-gauge needle without sonographic guidance. The biopsy was performed more than 1 month prior to MR imaging to avoid artifacts related to early post biopsy changes. Liver biopsies were performed in segments V and VI in order to correlate with DWI and to avoid sampling errors. Liver biopsy was not performed on patients without liver disease. The liver biopsy findings were retrospectively evaluated by an experienced pathologist. The Knodell histology activity index (HAI) for the grade of necroinflammatory changes and Metavir scoring system for the stage of liver fibrosis were used to record the severity of the disease. HAI system includes three subcategories: periportal necrosis and inflammation, scored from 0 to 10; intralobular necrosis and inflammation, scored from 0 to 4; and portal inflammation, scored from 0 to 4 (28). Fibrosis was staged on a 0-4 scale as follows: F0 – no fibrosis; F1 – portal fibrosis without septa; F2–portal fibrosis and few septa; F3–numerous septa without cirrhosis; and F4 – cirrhosis (29, 30). 

### 3.3. Imaging Protocol

**Figure 1.**
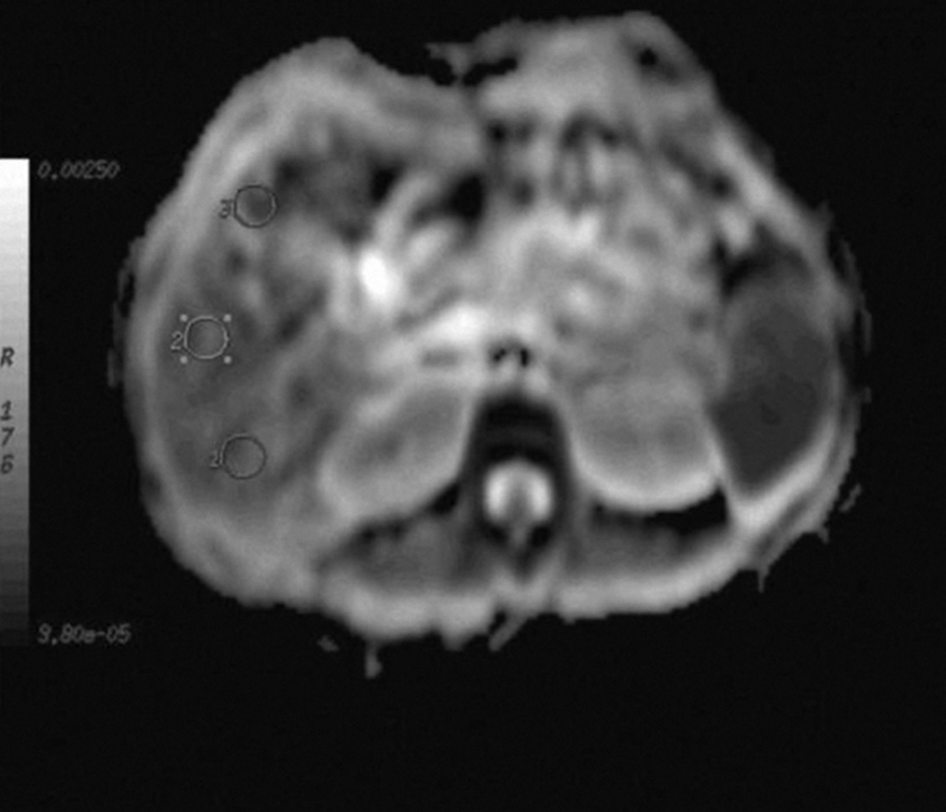
A 38-year-old patient with hepatic fibrosis. Sequence parameters: TR: 5000 ms, TE: 52 ms, b-value: 500 sec/mm ^2^. Measurement technique of ADC with 3 ROI is seen.

All patients were examined with a 1.5 T MRI unit (Signa Excite; GE Medical Systems, Milwaukee, Wisconsin, USA) using the standard body coil. The patients underwent DWI with a routine hepatic MRI protocol. The hepatic protocol included axial T1-weighted spin-echo (160 ms/1.8 ms, repetition time [TR]/echo time [TE]; number of excitations [NEX], 1.0; 10 mm slice thickness; 34×48 cm field of view [FOV]; 256×128 matrix), coronal T1-weighted fast spoiled gradient-echo sequence (160ms/1.8ms, TR/TE; NEX, 1.0; flip angle, 80 ^o^; 8 mm section thickness; 38×48 cm FOV; 256×128 matrix) with spectral fat saturation, axial T1-weighted fast spoiled gradient-echo sequence (160ms/1.8 ms, TR/TE; NEX, 1.0; flip angle, 80 ^o^; 10 mm section thickness; 34×48 cm FOV; 256×128 matrix) with spectral fat saturation obtained before and after intravenous contrast agent administration at arterial, portal and venous phases, axial T2-weighted fast spin-echo sequence (6000 ms/93ms, TR/TE; NEX, 2.0; 10 mm section thickness; 34×48 cm FOV; 512×256 matrix), coronal T2-weighted fast spin-echo sequence (6300 ms/130ms, TR/TE; NEX, 2.0; 7 mm section thickness; 38×48 cm FOV; 512×256 matrix). Spin echo echo-planar DWI was performed using the following acquisition parameters: TE=52-70 ms, TR=10000 ms, matrix=64×128, FOV=34×48, bandwidth=62-250kHz. Four different *b*-values were tested: 250, 500, 750 and 1000 sec/mm ^2^. A unidirectional (anterior-posterior) diffusion gradient was applied. Fat suppression was used to avoid image artifacts from subcutaneous fat. The patients and healthy volunteers held their breath for five seconds during expiration to avoid respiratory motion. Calculation of ADC values was performed on a workstation using the GE software functool – ADC. Three region of interests (ROI) were placed over the liver within segments V and VI in order to avoid motion artifacts and artifacts from the great vessels. The ROIs were manually and carefully positioned in the same region between two corresponding DWIs of different b factors and care was taken to ensure that the ROIs did not encompass the main blood vessels (Figure 1). Insufficient measurements were available in the left lobe owing to the effects of cardiac motion. From these 3 ROIs, the software calculated 3 ADC values and the mean value was used for statistical analysis. 

### 3.4. Statistical Analysis

Statistical analyses were performed with SPSS 19.0 program. The nonparametric Mann Whitney U-test was used in order to analyze the differences in skewed continuous variables, while differences in normally distributed continuous variables were compared by unpaired Student’s t-test. Multiple comparisons were assessed by analysis of variance (ANOVA) followed by Bonferroni’s correction and Kruskall-Wallis test followed by the Mann-Whitney U-test. Spearman correlation coefficients were used to test the correlation between fibrosis score (F1, F2, and F3) and *b*-values (250, 500, 750, 1000). A p-value less than 0.05 was considered significant. Data are presented as mean ± standard deviation.

## 4. Results

The histopathological characteristics of our 24 patients are summarized in Table 1. No patient had grade 4 inflammation, nor stage 4 fibrosis. All of the patients without liver disease assumed to have stage 0 fibrosis and grade 0 inflammation. There was a decrease in calculated ADC values for the patients diagnosed with hepatic fibrosis compared to patients without liver disease. There was a trend toward decrease in hepatic ADC values with an increasing degree of fibrosis. For each *b*-value employed in this study (250, 500, 750, and 1000 sec/mm ^2^), the mean hepatic ADC values of patients with hepatic fibrosis were significantly lower than those of patients without hepatic fibrosis (Table 2). 

**Table 1.** Histopathological Characteristics of 24 Patients

Characteristics	Frequency (Total Number of Patients=24)
**Cause of liver disease**	
HCV	15 (62.5%)
HBV	8 (33.3%)
HBV+HDV	1 (4.16%)
**Histologic activity index (Knodell)**	
0	0
1	1 (4.16%)
2	11 (45.8%)
3	12 (50.0%)
4	0
**Fibrosis score (Metavir)**	
F0	0
F1	10 (41.6%)
F2	9 (37.5%)
F3	5 (20.8%)
F4	0

**Table 2.** Comparison of Mean ADC Values of Liver Parenchyma in Patients With and Without Hepatic Fibrosis in Different b-Values

b- Value	ADC in Patients With Hepatic Fibrosis (mean±SD)(mm^2^/sec)	ADC in Patients without Hepatic Fibrosis (mean±SD)(mm^2^/sec)	P- Value
250	0.58×10^-3^±0.14×10^-3^	1.44×10^-3^±0.19×10^-3^	<0.01
500	0.77×10^-3^±0.16×10^-3^	1.33×10^-3^±0.16×10^-3^	<0.01
750	0.61×10^-3^±0.14×10^-3^	1.27×10^-3^±0.12×10^-3^	<0.01
1000	0.83×10^-3^±0.52×10^-3^	1.19×10^-3^±0.12×10^-3^	<0.01

**Figure 2.**
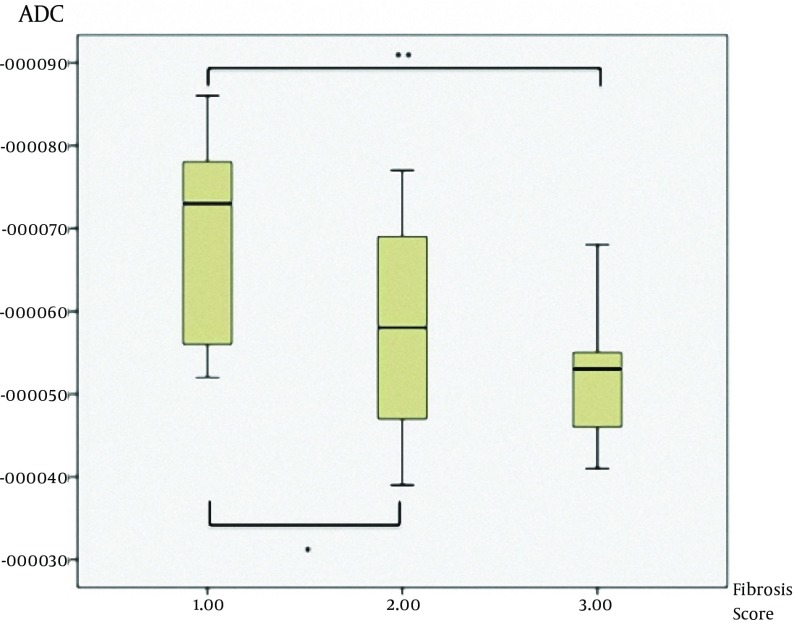
Differences of ADC values between F1, F2, and F3 groups at a b-value of 750 (*P=0.045 and **P=0.027)

**Figure 3.**
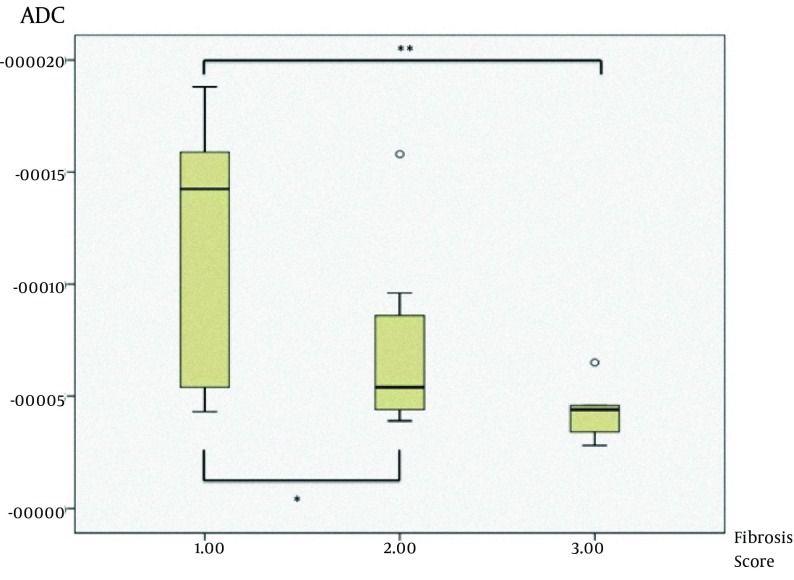
Differences of ADC values between F1, F2, and F3 groups at b-value of 1000 (*P=0.023 and **P=0.02)

As we mentioned above, we also evaluated the stage of hepatic fibrosis with Metavir scoring system. There were three subgroups of liver fibrosis stage according to Metavir scoring system (Table 1). We had no patient with stage 4 fibrosis (F4). We also compared the ADC values for all *b*-values between different hepatic fibrosis stages according to Metavir scoring (F1, F2, and F3). First, we found significant statistical difference in multivariate analysis among the whole group (P=0.025). Subsequently, we compared each ADC value at each *b*-value between F1, F2, and F3 with appropriate post-hoc tests (Table 3) to find out the difference between fibrosis score groups. In the post-hoc analysis, there were statistically significant differences between F1 and F2 (68.1×10^-3^±12.14×10^-3^ vs. 58.44×10^-3^±13.92×10^-3^, P=0.045) and F1 and F3 (68.1×10^-3 ^±12.14×10^-3 ^vs. 52.6×10^-3^±10.26×10^-3^, P=0.027) at a *b*-value of 750 (Figure 2), and also in the *b*-value of 1000, there were statistically significant differences between F1 and F2 (116.2×10^-3^±57.14×10^-3^ vs. 69.0×10 ^-3^±39.06×10^-3^ , P=0.023) and F1 and F3 (116.2×10^-3^±57.14×10^-3^ vs. 43.4×10^-3^ ±14.13×10^-3^, P=0.02) (Table 3) (Figure 3). 

**Table 3.** Comparison of Each ADC Value at All b-Values Between Fibrosis Stages F1, F2, and F3

	Fibrosis Score		
b - Value	F1	F2	F3
**250**	52.8×10^-3^±10.71×10^-3^	66.66×10^-3^±14.72×10^-3^	52.8×10^-3^±14.73×10^-3^
**500**	73.2×10^-3^±10.84×10^-3^	87.55×10^-3^±15.02×10^-3^	67×10^-3^±18.49×10^-3^
**750**	68.1×10^-3^±12.14×10^-3^	58.44×10^-3^±13.92×10^-3^	52.6×10^-3^±10.26×10^-3^
**1000**	116.2×10^-3^±57.14×10^-3^	69×10^-3^±39.06×10^-3^	43.4×10^-3^±14.13×10^-3^
**Post hoc analysis P-Values**			
**250 **	F1 vs F2; p=0.1		
	F1 vs F3; p=0.9		
	F2 vs F3; p=0.2		
**500**	F1 vs F2; p=0.5		
	F1 vs F3; p=0.2		
	F2 vs F3; p=0.6		
**750**	F1 vs F2; p=0.045		
	F1 vs F3; p=0.027		
	F2 vs F3; p=0.3		
**1000**	F1 vs F2; p=0.023		
	F1 vs F3; p=0.02		
	F2 vs F3; p=0.1		

**Figure 4.**
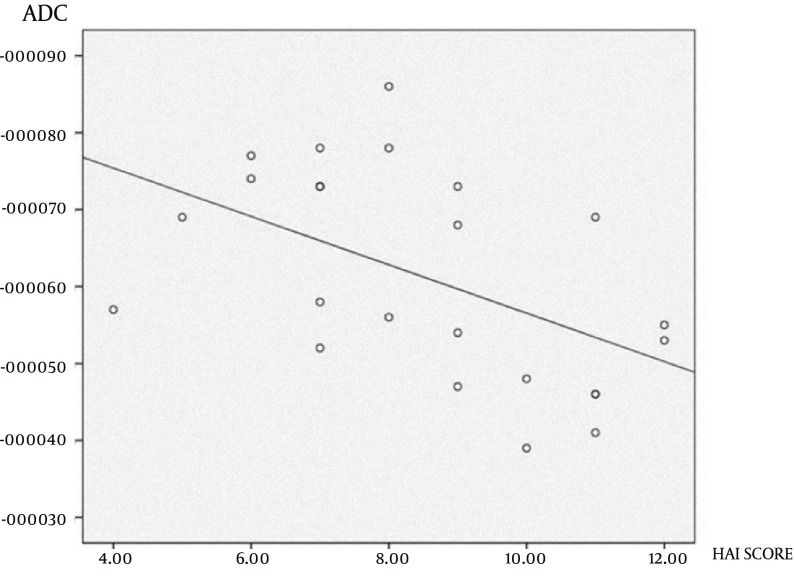
Correlation between HAI values and ADC values in a b-value of 750 sec/mm ^2 ^

Regarding patients with hepatic fibrosis, the correlation between HAI values and ADC values in a *b*-value of 750 sec/mm^2^ was significant, negative and moderate (r=-0.512; P=0.01) (Figure 4). For other *b*-values, the correlation with the HAI value was not significant. The correlations between the fibrosis score and ADC values in *b*-values of 750 and 1000 sec/mm^2^ were significant, negative, and moderate (r=-0.510; P=0.01 and r=-0.567; P=0.004, respectively) (Table 4). 

**Table 4.** Correlation Between ADC Values and HAI Scores or Fibrosis Scores for Different b-Values

b -Value	HAI Score (Correlation Coefficient, P-value)	Fibrosis Score (Correlation Coefficient, P-Value)
**250**	0.03; P>0.05	0.16; P>0.05
**500**	-0.15; P>0.05	-0.02; P>0.05
**750**	-0.512, P=0.01	-0.51; P=0.01
**1000**	-0.32; P>0.05	-0.567; P=0.004

## 5. Discussion

Clinical examinations and laboratory results are unreliable for differentiating the stages of hepatic fibrosis (22). The diagnosis of fibrosis stage 2 or greater is clinically important because owing to cost, risk of toxicity, and limited efficacy, only patients with fibrosis stage 2 or greater should receive antiviral treatment (31). MRI has become an important modality for assessing chronic liver disease. In a study carried out by Semelka et al. (32), two parenchymal enhancement patterns of chronic hepatitis were described using contrast enhanced dynamic MRI: early patchy enhancement indicating inflammatory changes in the liver and late linear enhancement indicating the presence of fibrosis (32). Cross-sectional imaging findings of advanced chronic hepatitis and cirrhosis are generally capable of detecting advanced diseases on the basis of signs of portal hypertension with good sensitivity and specificity (33). However, these findings have limited value in the detection of fibrosis (4). DWI has become possible in the abdomen with the advent of echoplanar MRI technique because it allows fast imaging and could minimize the effect of gross physiologic motion from respiration and cardiac movement (4, 6, 34). DWI is related to the diffusion of protons within tissues (35). Collagen fiber is the main component of hepatic fibrosis. The protons contained in this tissue are less abundant than those in water and are tightly bound (36). Therefore, diffusion in hepatic fibrosis should be restricted and the ADC values decreased compared to normal liver parenchyma (1). This statement was also confirmed by our results since we have demonstrated that ADC values within fibrotic liver parenchyma were reduced compared to ADC values within the normal parenchyma of patients without liver disease. Several studies have shown that the ADC of cirrhotic liver is lower than that of the normal liver, possibly due to the presence of a larger amount of connective tissue in the liver, narrowed sinusoids, and decreased blood flow (8, 37). There are limited data, however, on the correlation between hepatic ADC and the histologic stage of fibrosis.

Muller et al. (38) were the first to observe a reduced ADC in cirrhosis (using *b*-values up to 400 sec/mm^2^) (38). Amano et al. (39) reported that the difference of ADC values between cirrhotic and normal livers was greater in higher *b*-values (up to 400 sec/mm^2^) (40). Aube et al. (1) measured significant differences between healthy and cirrhotic livers (at a *b*-value of 200 sec/mm^2^) (1). DWI sequences using a low *b*-value were mostly sensitive to parenchymal microperfusion (41). A higher *b*-value must be used to increase the sensitivity to diffusion and to lessen the impact of perfusion. It therefore seems logical that improved results may be obtained by using higher *b*-values (250 to 1000 sec/mm^2 ^in our study). However, increasing the *b*-value results in a decreased signal. Therefore, calculated ADC values are decreased proportionally by the increase in *b*-value (42). In this respect, the choice of *b*-value plays a critical role. This value is a function of the amplitude and duration of the diffusion gradient and of the time allowed for the proton to diffuse between the 2 successive gradient pulses. Therefore, its choice is a compromise between adequate diffusion strength and image quality (4, 25, 42, 43).

A study, by Boulanger et al. (6) using 5 different *b*-values ranging from 50-250 sec/mm^2^, reported a lack of a significant difference between the control subjects and HCV patients (6). In another study performed by Koinuma et al. (22), *b*-values of 0 and 128 sec/mm^2 ^were used and a correlation between ADC values and fibrosis scores were detected while no correlation between ADC and inflammation grade was found (22). Both studies involved relatively small *b*-values that could not detect the differences between fibrotic and nonfibrotic liver; because the diffusion signal intensity was contaminated by perfusion (22, 42, 43). The study conducted by Hollingsworth et al. (5) suggested that in order to limit the influence of perfusion changes, breath-hold diffusion weighted studies of the liver should be performed at high *b*-values (750 and 500 sec/mm^2^) (5).

In our study, we used 4 different *b*-values (250, 500, 750 and 1000 sec/mm^2^) in order to obtain a more precise calculation of ADC with less perfusion contamination and less regional ADC variation. The results showed that ADC values decreased as the stage of liver disease progressed from normal function to chronic hepatitis. The relationship between ADC values and the fibrosis score was significant with the use of higher *b*-values (750 and 1000 sec/mm^2^). We also found a significant relationship between ADC values and necroinflammation scores with the use of a 750 sec/mm^2^ *b*-value.

Transient elastography, real-time elastography and MR elastography are other new techniques that can be used in non-invasive evaluation of liver fibrosis. Transient elastography is a rapid and reproducible technique equipped with a probe consisting of an ultrasonic transducer mounted on the axis of a vibrator (44, 45). The liver stiffness is automatically calculated from the velocity of propagation of an elastic shear wave through the liver parenchyma that is induced by vibrations transmitted toward the tissue. The stiffness of the liver is recorded in kilopascal (kPa) (45-47). Assessment of different blood markers using transient elastography measurement of tissue elasticity has shown hopeful results in determining the degree of liver fibrosis (48). In a previous study by Lewin et al. (4), out of 54 hepatitis C patients, the ADC values of 23 patients (with fibrosis stages of F2 and F3) were compared based on their elastography, FibroTest, aspartate aminotransferase to platelets ratio index (APRI), Forns index, and hyaluronate results. Consequently, it was stated in this study that in cases where liver fibrosis is evident, DWI is more useful than other non-invasive techniques in detecting the degree of fibrosis. It is also stated that the combination of ADC and transient elastography resulted in the best diagnostic performance for significant fibrosis (F≥2) (4).Transient elastography can be an ineffective method in case of obesity, narrow intercostal space and ascites. In a study by Fraquelly et al. (49) an overall 2.4% rate of indeterminate results of transient elastography was noted that was due to high body mass index (BMI>28 kg/m^2^) in four patients and narrow intercostal space in one patient (49). Real-time elastography is an ultrasound-based method to measure tissue elasticity and it is technically different from transient elastography. With conventional ultrasound probes, echo signals before and under slight compression are compared and analyzed (48, 50). In contrast to transient elastography, this method can also be used effectively in case of unfavorable conditions such as patient obesity and ascites.

MR elastography is a promising new non-invasive MR imaging technique that quantifies the stiffness of the tissues. In this technique, MR images are obtained with a gradient-echo sequence as the waves propagate through the liver. Liver stiffness measured with MR elastography increases as the stage of fibrosis advances (36).

There are several studies about the potential applications of these non-invasive methods; in particular, transient elastography, for evaluation of liver fibrosis. In a study by Kim et al. (51), transient elastography was performed in patients with chronic hepatitis B infection during long-term antiviral treatment. They aimed to assess liver fibrosis regression in these patients (51). Furthermore, there are studies in which liver stiffness measurements are found to be effective in predicting clinical decompensation and portal hypertension-related complications such as esophageal varices in patients with chronic liver disease (52-55). Transient elastography has also been used to assess the degree of liver fibrosis and the risk of hepatocellular carcinoma development in patients with chronic hepatitis (56, 57).

Our study had several limitations. One of which was the inability to use a surface coil for the acquisition of DWI data. The use of body coil significantly decreases the acquired signal and signal-to-noise ratio (SNR) (1, 39). The use of surface coils, by increasing the amount of acquired signal, should provide significant results using higher *b*-values (>400 sec/mm^2^) (1). Other limitations to our study include the small number of subjects and intermediate levels of hepatic fibrosis and necroinflammation, limiting the ability to achieve statistically significant results. Future work is needed to assess larger numbers of patients and to correlate DWI findings with findings obtained with newer methods of perfusion MRI (58, 59), MR elastography (60, 61) and serologic markers of fibrosis.

In conclusion, our findings suggest that hepatic ADC measurement with DWI with a *b*-value of 750 sec/mm^2 ^or greater can be used in accurate quantification of liver fibrosis and necroinflammation. DWI can be used as an adjunct to the routine MRI protocol. It may be possible to use DWI findings for follow up of patients with chronic hepatitis.
